# Optimizing the Properties of La_0.8_Sr_0.2_CrO_3_ Thin Films through Post-Annealing for High-Temperature Sensing

**DOI:** 10.3390/nano11071802

**Published:** 2021-07-11

**Authors:** Dan Liu, Peng Shi, Yantao Liu, Yijun Zhang, Bian Tian, Wei Ren

**Affiliations:** 1 Key Laboratory of Instrumentation Science & Dynamic Measurement, Ministry of Education, North University of China, Taiyuan 030051, China; 2 Electronic Materials Research Laboratory, Key Laboratory of the Ministry of Education & International Center for Dielectric Research, School of Electronic and Information Engineering, Xi’an Jiaotong University, Xi’an 710049, China; zhangyj518@mail.xjtu.edu.cn (Y.Z.); wren@mail.xjtu.edu.cn (W.R.); 3 Department of Electronic Engineering, Xi’an University of Technology, Xi’an 710048, China; liuytxjtu@163.com; 4 State Key Laboratory for Mechanical Manufacturing Systems Engineering, School of Mechanical Engineering, Xi’an Jiaotong University, Xi’an 710049, China; t.b12@mail.xjtu.edu.cn

**Keywords:** post-annealing, La_0.8_Sr_0.2_CrO_3_, thin-film thermocouple, high-temperature sensing

## Abstract

La_0.8_Sr_0.2_CrO_3_ (0.2LSCO) thin films were prepared via the RF sputtering method to fabricate thin-film thermocouples (TFTCs), and post-annealing processes were employed to optimize their properties to sense high temperatures. The XRD patterns of the 0.2LSCO thin films showed a pure phase, and their crystallinities increased with the post-annealing temperature from 800 °C to 1000 °C, while some impurity phases of Cr_2_O_3_ and SrCr_2_O_7_ were observed above 1000 °C. The surface images indicated that the grain size increased first and then decreased, and the maximum size was 0.71 μm at 1100 °C. The cross-sectional images showed that the thickness of the 0.2LSCO thin films decreased significantly above 1000 °C, which was mainly due to the evaporation of Sr^2+^ and Cr^3+^. At the same time, the maximum conductivity was achieved for the film annealed at 1000 °C, which was 6.25 × 10^−2^ S/cm. When the thin films post-annealed at different temperatures were coupled with Pt reference electrodes to form TFTCs, the trend of output voltage to first increase and then decrease was observed, and the maximum average Seebeck coefficient of 167.8 µV/°C was obtained for the 0.2LSCO thin film post-annealed at 1100 °C. Through post-annealing optimization, the best post-annealing temperature was 1000 °C, which made the 0.2LSCO thin film more stable to monitor the temperatures of turbine engines for a long period of time.

## 1. Introduction

The gas turbine engine at the heart of the airplane is a highly complex and precise thermal machinery, where its working temperature needs to be monitored in real time and in situ to reflect the operational conditions for designing constructions or warning states of modern propulsion systems; however, it is difficult to obtain operating temperatures accurately with available technologies due to the extreme conditions [1]. Various noncontacting sensing technologies have been employed, such as optic pyrometers [2] and acoustic pyrometers [3], but the measuring temperature of turbine engines is not direct and accurate, due to the principles of these technologies, and their complicated structures and sensing modules do not make them easy to integrate with the turbine engine. However, thin-film thermocouples (TFTCs) as a typical kind of immersive sensor can be deposited directly onto the surfaces of turbine engine components with a thickness of a few micro/nanometers using modern deposition technologies; they have been promising as thermal sensors for turbine engines due to the advantages of excellent spatial resolution, cost-effective in large quantities, and rapid response [4,5,6,7,8].

TFTCs consist of two beams that should have excellent oxidation resistance and chemical stability because of the high working temperature in air, and the two beams should have large and stable Seebeck coefficients to form a TFTC with higher sensitivity. It is well-known that a wire-type thermocouple fabricated by conventional noble metals, such as Pt, Rh, or their alloys, can be used to measure high temperatures in air, which can even endure up to about 1820 °C with Al_2_O_3_ shield protection [9]. Conversely, the maximum measurement temperature is only 1100 °C when it is changed into a thin film, which is mainly ascribed to the coalescence reaction between the TFTC and substrate, and the oxidation of rhodium above 800 °C [10,11]. In addition, its sensitivity is lower due to the small intrinsic Seebeck coefficients of noble metals. Therefore, the development of a new TFTC fabricated by conductive ceramics has been a trend to measure high temperatures in air [12,13,14,15]. Especially, conductive oxides with better oxidation resistance and higher Seebeck coefficients have become the most promising candidate materials of electrodes for TFTCs, such as In_2_O_3_, ITO, ZnO:Al (AZO), and La_1-x_Sr_x_CrO_3_ (xLSCO) [14,16,17,18,19]. When In_2_O_3_ and ITO were used to fabricate the electrodes of TFTCs, they had excellent performances with a maximum measurement temperature of 1280 °C, while a serious volatilization problem appeared for them above 1200 °C especially because of the losing of Sn^4+^ from ITO, and the indium element is scarce in the earth [20,21,22]. Meanwhile, for xLSCO, wider applications in the energy and sensor fields of the present materials in the thin films field have also been reported in recent years. They are suitable for interconnect materials in solid oxide fuel cells [23,24,25], transparent electrolytes and electrodes of batteries and generators [26,27,28,29,30], thin-film fluorescent sensors, and high-temperature sensing [17,18,31,32], with their high melting point of 2490 °C, good conductivity, and high Seebeck coefficient. From our previous work [17], a screen-printed thick-film thermocouple fabricated by LaCrO_3_ (LCO) and La_0.8_Sr_0.2_CrO_3_ can be used to measure a temperature of 1550 °C for 10 h in air, which has inspired the fabrication of TFTCs by using xLSCO electrodes. Since then, some TFTCs fabricated by the 0.2LSCO thin film have been investigated, such as 0.2LSCO-In_2_O_3_ [19] and 0.2LSCO-Pt [33], with a higher sensitivity and stable output voltage versus the temperature difference. However, the properties of 0.2LSCO thin films can be affected significantly by the post-annealing treatment process, which have not yet been investigated systematically and should be studied to fabricate TFTCs with a stable output voltage curve to use at high temperatures.

In this paper, 0.2LSCO thin films were fabricated successfully via the RF sputtering method. The effects of different post-annealing temperatures on the microstructures, morphologies, and electrical conductivities of 0.2LSCO thin films were investigated systematically. Then, the thermoelectric properties of 0.2LSCO thin films were studied by coupling with platinum (Pt) reference electrodes at a static calibration system. Finally, the optimal post-annealing temperature was obtained by comprehensive analysis.

## 2. Experimental Sections

A La_0.8_Sr_0.2_CrO_3_ target was used to deposit the 0.2LSCO thin films onto alumina substrates with sizes of 100 mm × 20 mm × 1 mm via the RF sputtering method. A JDP-560 model sputtering system (Sky Technology Development Co., Ltd., Shen yang, China) was used to deposit the thin films. The detailed deposition processes were the same as our previous work [33]. Before the deposition of the thin films, the alumina substrates were cleaned by ultrasonication with acetone, ethyl alcohol, and deionized water, successively. Then, the 0.2LSCO thin films were deposited on cleaned alumina substrates by the RF sputtering system at 500 °C. Finally, the as-deposited thin films were post-annealed at different temperatures from 800 °C to 1300 °C for 1 h with an interval of 100 °C.

A Dmax/1400 X-ray diffractometer (Rigaku, Tokyo, Japan, Cu Kα radiation) was used to characterize the crystal structures of 0.2LSCO thin films with a step of 0.02°. The surface and cross-sectional SEM images were obtained by a FEI Quanta 250 FEG field-emission scanning electron microscope (FEI, Hillsboro, OR, USA), and the composition of elements in the thin films was analyzed by an energy-dispersive spectrometer. The conductivities of 0.2LSCO thin films were measured by the four-point probe method using the Vanderbilt law.

To characterize the thermoelectric properties of the 0.2LSCO thin films, they were sputtered to form L patterns using the photolithography technique, and then post-annealed at different temperatures for 1 h. Subsequently, another electrode of Pt thin films was coupled with 0.2LSCO thin films to form TFTCs with a U-pattern. Finally, the fabricated TFTCs were pasted with copper wires at the cold end using silver paste, and then heated at 200 °C for 1 h to make the bonds strong. The output voltages of the TFTCs were measured by a lab-made testing measurement system, similar to our previous work [20]. A muffle furnace (LHT 02/17/P310, Nabertherm, Lilienthal, Germany) was used to provide various temperature differences. The temperature of the hot-junction was collected by a Type-S thermocouple, and the temperature of the cold-end was measured by a Type-K thermocouple at the same time. In order to maintain the reproducibility of the measurement system, the cold-end was chilled and the temperature was kept at 25 °C with circulating water during the experiments, while the hot-junction was fixed in the same place in the furnace. The data of temperatures and output voltages were recorded simultaneously by using a USB Data Acquisition system (LR8431-30, HIOKI, Nagano-ken, Japan) equipped with “Logger Utility” software.

## 3. Results

**Figure 1:**
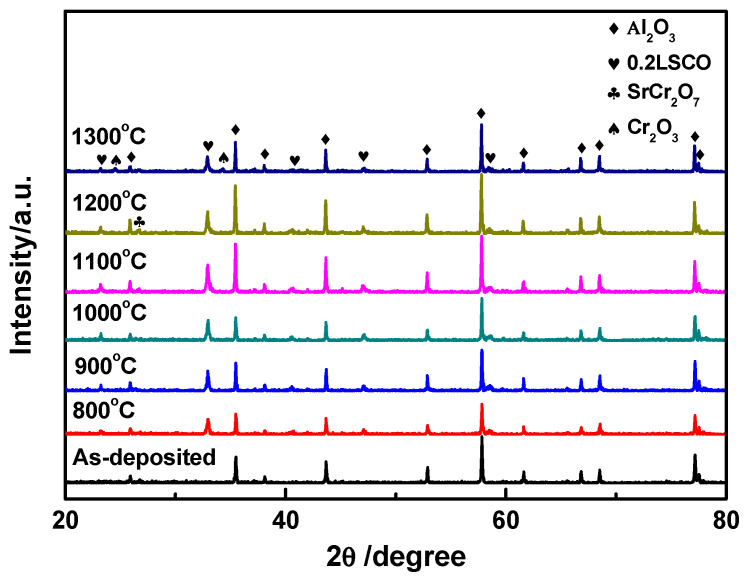
XRD patterns of 0.2LSCO thin films at different post-annealing temperatures.

To withstand high temperatures for a long time, the 0.2LSCO thin films were deposited for 4 h by using a sputtering system (where the RF power was 150 W, the target diameter was 101.6 mm, the gas pressure was 1.3 Pa, and the O_2_/Ar ratio was 0.13/1.17), and then a post-annealing process was carried out from 800 °C to 1300 °C for 1 h. The XRD patterns of different post-annealing temperatures for 0.2LSCO thin films are shown in Figure 1. For the as-deposited 0.2LSCO thin film, only the characteristic peaks of the alumina substrate can be observed, which shows that the film was amorphous. When the post-annealing temperature was 800 °C, the characteristic peaks of 0.2LSCO appeared, and its intensity increased with temperature from 800 °C to 1000 °C, which indicates that a higher temperature was beneficial for the crystallinity of the 0.2LSCO thin film. Conversely, when the post-annealing temperature increased to 1100 °C and 1200 °C, the peaks of impurity phases of Cr_2_O_3_ marked as spades were observed due to the volatilization of strontium lanthanum chromate above 1000 °C [34]. Moreover, the peaks of Cr_2_O_3_ disappeared and the peaks of SrCr_2_O_7_ were observed for the sample post-annealed at 1300 °C, which may be attributed to the secondary combination after the decomposition of the constituent elements for the 0.2LSCO thin film at high temperatures.

**Figure 2:**
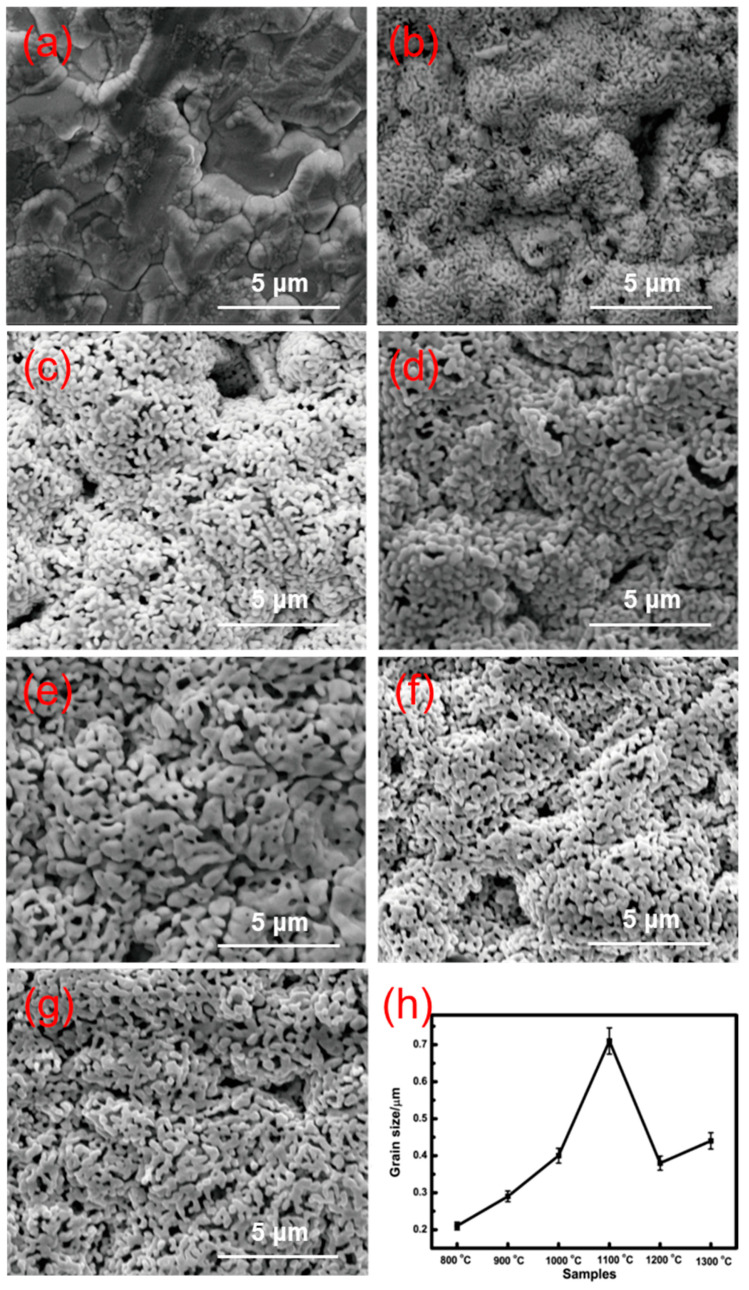
Surface morphologies of 0.2LSCO thin films annealed at different temperatures: (**a**) deposited; (**b**) 800 °C; (**c**) 900 °C; (**d**) 1000 °C; (**e**) 1100 °C; (**f**) 1200 °C; (**g**) 1300 °C; (**h**) the change in grain size with different post-annealing temperatures.

The surface morphologies of 0.2LSCO thin films post-annealed at different temperatures were measured, and their SEM images are shown in Figure 2a–e. The grain size of the films with different temperatures was obtained by means of the standard root-mean-square method from the SEM images, and the results are shown in Figure 2h. In Figure 2a, for the as-deposited 0.2LSCO thin film, the morphology mainly consisted of grains of the alumina substrate, and the surface of the alumina substrate was uneven, which was due to the amorphous structure of the 0.2LSCO thin film. When the post-annealing temperature reached 800 °C, the surface morphology of the 0.2LSCO thin film was totally different from its as-deposited stage, and a lot of tiny spindle structures could be observed, as shown in Figure 2b, which indicates that the 0.2LSCO thin film was not crystalline. With the increases in post-annealing temperature, the grain size of the 0.2LSCO thin film increased significantly from 0.2 μm at 800 °C to 0.71 μm at 1100 °C, as shown in Figure 2h.

**Figure 3:**
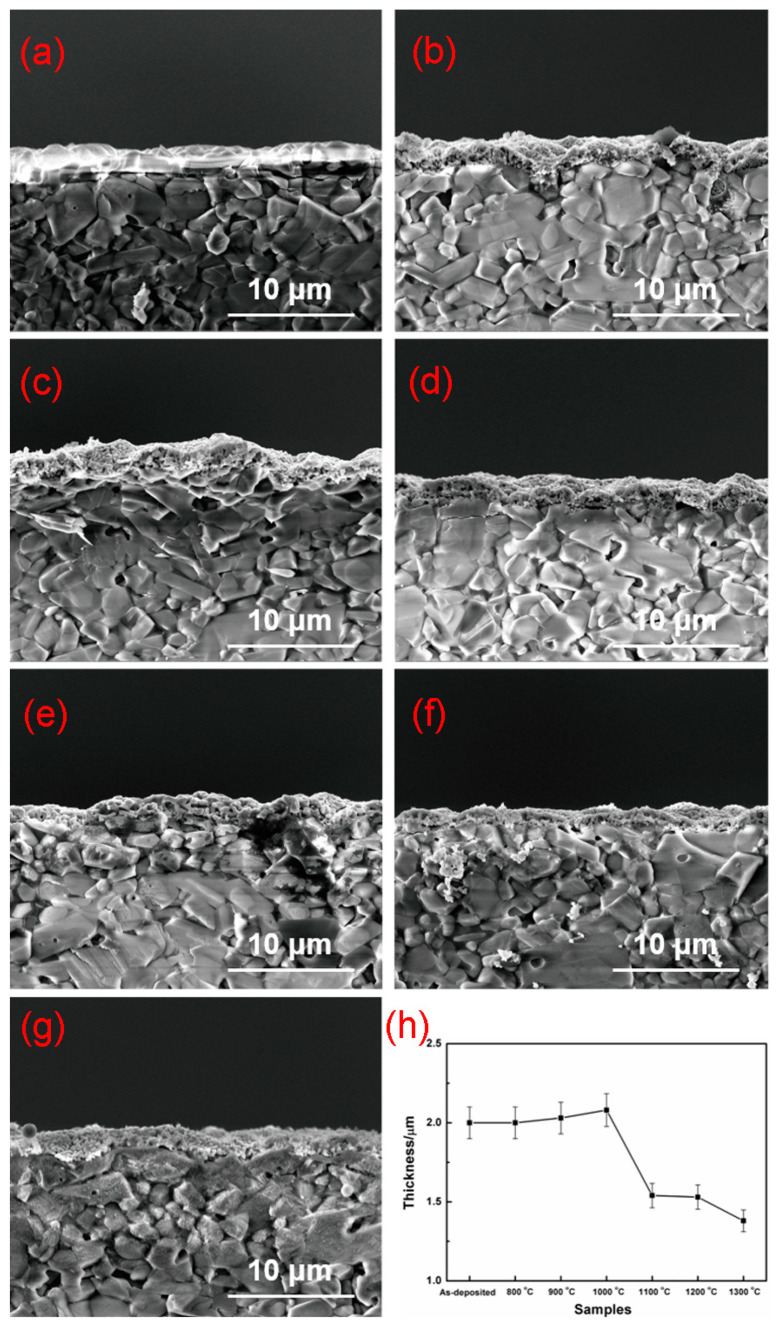
Cross-sections of 0.2LSCO thin films annealed at different temperatures: (**a**) as-deposited; (**b**) 800 °C; (**c**) 900 °C; (**d**) 1000 °C; (**e**) 1100 °C; (**f**) 1200 °C; (**g**) 1300 °C; (**h**) the change in thickness.

The SEM images of cross-sections at different annealing temperatures are shown in Figure 3. The statistical average thicknesses of 0.2LSCO thin films post-annealed at different temperatures were measured from the SEM images, and the changing curve of thicknesses is shown in Figure 3h. For the as-deposited 0.2LSCO thin film, its thickness was about 2.0 μm. The thickness increased slightly with the annealing temperature and reached up to the maximum value of 2.08 μm at 1000 °C, which was mainly ascribed to volume expansion induced by the increase in grain size, as shown in Figure 2. However, the thickness decreased significantly when the annealing temperature was higher than 1100 °C. The thickness of the 0.2LSCO thin film changed from 1.54 μm at 1100 °C to the minimum value of 1.38 μm at 1300 °C, which decreased about 31% compared with that of the as-deposited sample.

**Figure 4:**
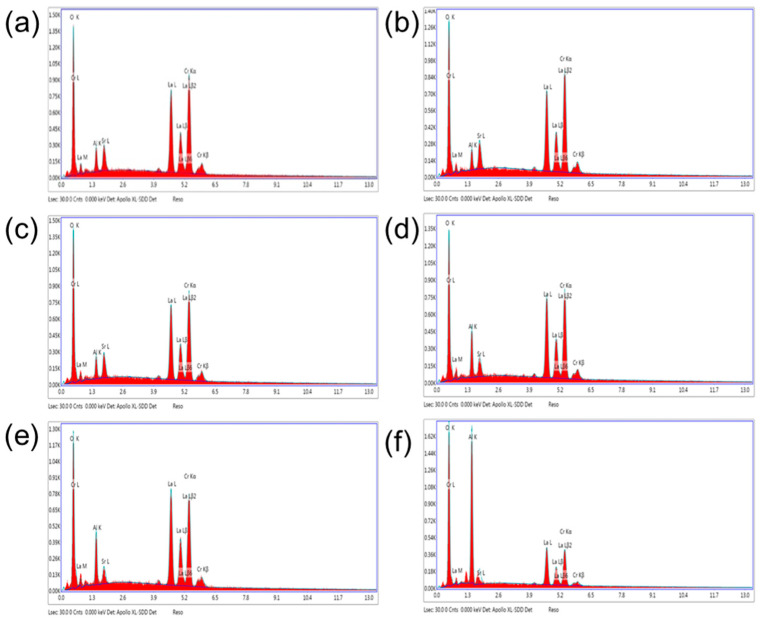
EDS results of 0.2LSCO thin films annealed at different temperatures for 1 h: (**a**) 800 °C; (**b**) 900 °C; (**c**) 1000 °C; (**d**) 1100 °C; (**e**) 1200 °C; (**f**) 1300 °C.

The constituent elements of the 0.2LSCO thin films post-annealed at different temperatures were measured by EDAX, and the results are shown in Figure 4. Here, the main elemental content of Al, O, La, Sr, and Cr was chosen to analyze the effect of different post-annealing temperatures, and the results are listed in Table 1. The contents of Sr and Cr remained nearly unchanged below 1000 °C, and they then decreased above 1100 °C. For the element La, its content changed slightly even at the post-annealing temperature of 1200 °C, which showed that the evaporation of the 0.2LSCO thin film was mainly in the form of compounds containing elements of Sr and Cr. However, the contents of Sr, La, and Cr decreased significantly for the sample post-annealed at 1300 °C. It is also interesting to note that the content of the Al element had an increasing tendency with the increase in temperature, due to the intensity of aluminum oxide detected with the decreases in the films’ thickness.

**Figure 5:**
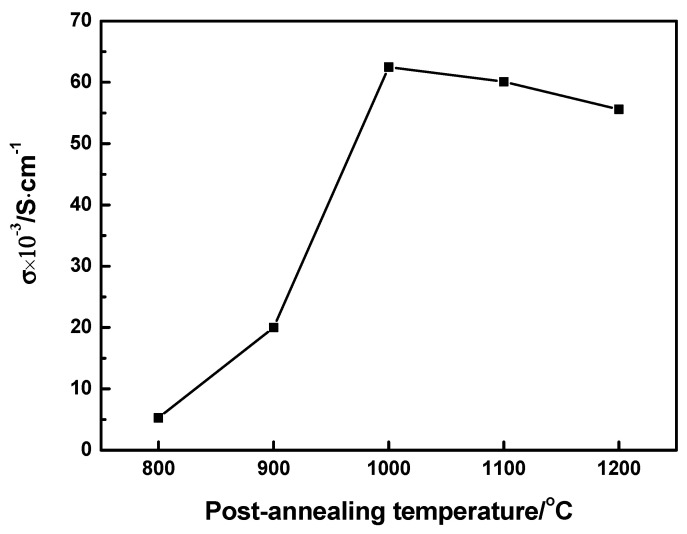
Conductivities of 0.2LSCO thin films at different post-annealing temperatures.

Figure 5 shows the conductivities of 0.2LSCO thin films annealed at different temperatures using the four-point probe method of the Vanderbilt law. There was a tendency of the conductivity to rise first and then decrease, and the maximum value of 6.25 × 10^−2^ S/cm was obtained for the 0.2LSCO thin film annealed at 1000 °C. For the rising stage, the crystallinity and grain size of the 0.2LSCO thin film increased with the post-annealing temperature, which resulted in a decrease in the scattering among grain boundaries for carriers. Conversely, for the decreasing stage, the thermal volatilization and formation of the nonconductive secondary phase made the conductivity deteriorative and even insulative at 1300 °C. In other words, an appropriate post-annealing temperature will enhance the conductivity of the 0.2LSCO thin film and reduce the volatilization.

**Figure 6:**
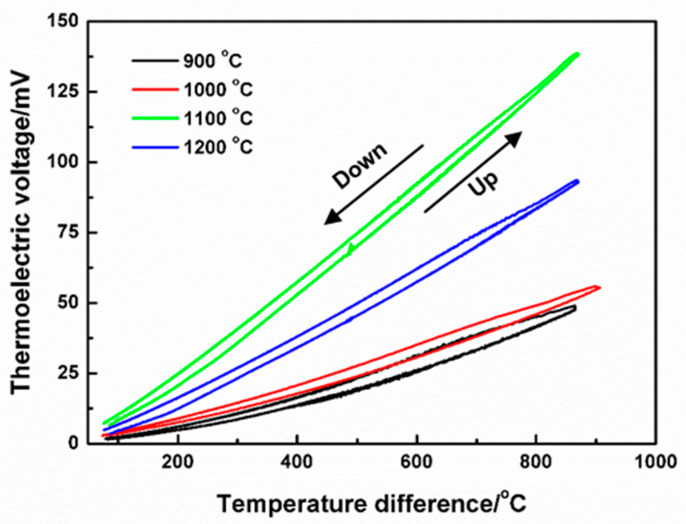
Thermoelectric voltages of 0.2LSCO-Pt TFTCs at different temperature differences.

The thermoelectric properties of 0.2LSCO thin films post-annealed at different temperatures were measured by forming U-type TFTCs with Pt thin-film electrodes using a static calibration, and the variation curves of thermal voltages with temperature differences are shown in Figure 6. For the 0.2LSCO thin film annealed at 800 °C, it was difficult to collect the thermal voltages from the voltmeter due to its poor conductivity and lower crystallinity, and its thermal voltage curve has not been marked in this figure. In addition, the thermal voltages have also not been marked for the 0.2LSCO thin film annealed at 1300 °C, because of the interruption of electrical transport of the Pt electrode. For others, their thermal voltages increased first and then decreased with the post-annealing temperature. The results of average Seebeck coefficients of the 0.2LSCO thin films at different post-annealing temperatures are listed in Table 2. The maximum thermal voltage of 167.8 µV/°C was obtained for the 0.2LSCO thin film annealed at 1100 °C. The reproducibility of the measurements in this lab-made system can be achieved by fixing the place of the hot-junction in the furnace, as well as the temperature at the cold-end, for each test.

## 4. Discussion

From the results of XRD and SEM for 0.2LSCO thin films annealed at different temperatures, the as-deposited 0.2LSCO thin film shows an amorphous structure and needs post-annealing to be suitable as electrodes of TFTCs. When the post-annealing temperature was lower than 800 °C, the amorphous structure was shown in the 0.2LSCO thin film and, accordingly, the crystallization was not good, which agrees with the research results of the literature [35]; a higher temperature over 780 °C should be taken to crystallize chromic acid lanthanum. With the post-annealing temperature increased from 800 °C to 1100 °C, the grain size of the 0.2LSCO thin film increased significantly from 0.2 μm at 800 °C to 0.71 μm at 1100 °C, while the thickness of the films remained stable at about 2.0 μm, indicating the good crystallization of the films. However, when the temperature was higher than 1100 °C, the grain size of the 0.2LSCO thin film decreased with the post-annealing temperature to 1300 °C, while the thickness decreased about 38% at the same time. For this reason, one is due to the emergence of the secondary phases and the intensification of volatilization of Cr_2_O_3_ and SrCr_2_O_7_ at higher temperatures; the other is due to the higher specific surface area of the material and the increase in Gibbs free energy, which enhances the reaction between the 0.2LSCO thin film and external thermal environment. Post-annealing treatment will change the morphologies of 0.2LSCO thin films and has an impact on their service stability for high temperatures. Therefore, the post-annealing temperature should be kept below 1100 °C through comprehensive analysis.

In order to obtain optimal parameters for the preparation of thin-film thermocouples, the thermoelectric response with different annealing temperatures was studied accordingly. The maximum thermal voltage of 167.8 µV/°C was obtained for the 0.2LSCO thin film annealed at 1100 °C. Furthermore, hysteresis phenomena of thermal voltages were observed for all TFTCs between heating and cooling processes because of defect concentration, crystal structure, grain size, and heating rate. On the other hand, it was caused by the formation of a small amount of secondary phase and the decrease in thickness. Conversely, for the sample annealed at 1200 °C, the thickness of the thin film reduced further due to the enhancement of evaporation, and lower thermal voltages were achieved.

It is well known that the principle of thermocouples is based on the Seebeck effect, which can be defined as Equation (1) [19]:

S = ∆V/∆T(1)

where S is the Seebeck coefficient, ∆V is the output voltage, and ∆T is the difference temperature between the hot end and cold end. By calculation, the S values of 0.2LSCO-Pt thin-film thermocouples were nearly the same as those of the 0.2LSCO thin films themselves due to the S of Pt being neglected (1.67 V/°C for standard Pt wire [36]). From our previous article [19], the 0.2LSCO thin film annealed at 1000 °C was very stable, and the draft rate of a 0.2LSCO/In_2_O_3_ TFTC was only 0.58 °C/h, which was almost equal to that of a standard type-K wire thermocouple (0.18 °C/h) with a diameter of 0.5 mm. At the same time, combining with the results of the crystal structure, morphology, and electrical conductivity mentioned above, the post-annealing temperature of 1000 °C was more beneficial for high-temperature sensing. In other words, the post-annealing treatment will affect the microstructure composition, volatilization, and thermoelectric output performance of the 0.2LSCO thin film, and a higher-sensitivity TFTC fabricated by the 0.2LSCO thin film can be achieved to sense high temperatures in air.

## 5. Conclusions

La_0.8_Sr_0.2_CrO_3_ thin films were prepared via the RF sputtering method and post-annealed at different temperatures. 0.2LSCO thin films with a pure phase were obtained and the intensity of XRD peaks increased with the temperature from 800 °C to 1000 °C, while impurity phases of Cr_2_O_3_ and SrCr_2_O_7_ appeared above 1000 °C. Meanwhile, the grain sizes of 0.2LSCO thin films had a tendency to increase first and then decrease, and the maximum size was 0.71 μm for the thin film post-annealed at 1100 °C due to the apparent melting and agglomerating phenomena. The thicknesses of 0.2LSCO thin films decreased significantly above 1000 °C due to the evaporation of Sr^2+^ and Cr^3+^. The conductivities of 0.2LSCO thin films increased first and then decreased, with a maximum value of 6.25 × 10^−2^ S/cm obtained for the post-annealing temperature of 1000 °C, while the thermal volatilization and the formation of a nonconductive secondary phase made the conductivity deteriorative at 1300 °C. By coupling with Pt reference electrodes to form TFTCs, the maximum average Seebeck coefficient of 167.8 µV/°C was achieved for the 0.2LSCO thin film annealed at 1100 °C. The results indicated that the properties of the 0.2LSCO thin film can be affected by post-annealing temperature significantly, and an appropriate treatment should be taken to make the 0.2LSCO thin film more stable to fabricate TFTCs for sensing at high temperatures.

## Figures and Tables

**Table 1:** Contents of main elements in 0.2LSCO thin films with different post-annealing temperatures.

Temperature (°C)	Element Atomic Ratio (%)				
	O	Al	Sr	La	Cr
800	55.23	6.12	2.76	15.4	20.48
900	57.46	5.14	2.96	12.96	21.48
1000	59.58	5.94	2.7	12.57	19.22
1100	55.24	11.53	2.22	13.06	17.96
1200	54.56	12.07	1.73	14.46	17.17
1300	61.26	25.12	1.42	5.53	6.67

**Table 2:** Average Seebeck coefficients of 0.2LSCO thin films at different post-annealing temperatures.

Post-Annealing Temperature (°C)	Seebeck Coefficient (µV/°C)
900	59.0
1000	62.2
1100	167.8
1200	114.1
